# Hematology and serum biochemistry variables in apparently normal Arabian Oryx (*Oryx leucoryx*)

**DOI:** 10.14202/vetworld.2023.1369-1372

**Published:** 2023-06-17

**Authors:** Abdulhakeem Eljarah, Zuhair Bani Ismail

**Affiliations:** Department of Veterinary Clinical Sciences, Faculty of Veterinary Medicine, Jordan University of Science and Technology, P.O. Box 3030, Irbid 22110, Jordan

**Keywords:** clinical examination, general health status, laboratory analysis, wildlife

## Abstract

**Background and Aim:**

Hematology and serum biochemical analyses are integral parts of the clinical evaluation of sick animals. This is especially true regarding the clinical care of wildlife species, where clinical signs and historical data relating to the particular illness are often not available. Therefore, this study was designed to report various hematology and serum biochemistry parameters in Arabian oryx (*Oryx leucoryx*).

**Materials and Methods:**

Various hematology and serum biochemistry parameters were determined in 49 Arabian oryx of various ages and sexes. Hematology parameters included total red blood cells (RBC), packed cell volume (PCV), hemoglobin concentration, mean corpuscular volume, mean corpuscular hemoglobin (MCH), MCH concentration, and total leukocyte count (white blood cell) using an automated hematology analyzer. Serum biochemistry variables included glucose (Trinder method), total protein (biuret method), albumin using the Bromcresol Green (BCG) method, and blood urea nitrogen (colorimetric method). In addition, serum electrolyte concentrations of calcium, magnesium, and phosphorus were determined using colorimetric methods.

**Results:**

There was a significant difference in RBC count, PCV, and serum glucose concentration between adult and young Arabian oryx. The RBC count was significantly higher in males than in females, whereas the serum glucose concentration was significantly higher in females.

**Conclusion:**

Results of this study showed significant differences in RBC, PCV, and serum glucose concentration between apparently normal young and Adult Arabian oryx. Similar differences were also detected between normal males and females. Knowledge of these data could prove vital in the clinical evaluation of the health status of this wildlife species.

## Introduction

Historically, 12 different species of ungulates have been reported from the wild in Jordan (*Addax nasomaculatus* (Addax), *Bos primigenius* (Aurochs), *Cervus elaphus* (Red Deer), *Dama mesopotamica* (Mesopotamian Fallow Deer), *Oryx leucoryx* (Arabian Oryx), *Equus hemionus hemippus* (Syrian Wild Ass), Wild Boar (*Sus scrofa*), *Gazella gazella* (Palestinian Mountain Gazelle), *Capreolus capreolus* (European Roe Deer), *Gazella marica* (Arabian Sand Gazelle), and *Gazella dorcas* (Dorcas Gazelle), and *Capra nubiana* (Nubian Ibex) [1, 2]. Unfortunately, Addax, Aurochs, and Red Deer have already been extinct, while the other nine species are endangered and currently are kept in conservation and managed in semi-captive conditions [1–10]. Since its reintroduction to its normal habitats in conservation by the Royal Society for the Conservation of Nature in 1978, the species has been thriving [1, 2].

The health management of this species is challenging due to its wild nature [1, 2]. The oryx are shy animals, and sick animals often do not show clear signs of illness, and many are found dead in their enclosures [11–13]. Although sick animals isolate themselves, capturing them is very stressful and requires skill and specialized equipment, making clinical assessment even more difficult [12]. Hematology evaluation and serum biochemical analyses have been used to determine the health status of domestic animals [1–3]. They may indicate various abnormal changes in the body, including stressful or painful responses, inflammatory and infectious processes, and physiologic and cellular changes involving vital organs such as the liver and kidneys [10]. The current literature concerning health care and disease diagnosis in oryx offers little information. Most of the available data do not cover all oryx species, including *Oryx leukoryx* [1–10].

Establishing normal or reference values for the hematology and serum biochemistry values in Arabian oryx would be the first step in understanding and elucidating the physiological adaptations of this species to the very arid conditions of its natural habitat. Therefore, this study aimed to determine the normal or reference ranges for the hematology and serum biochemistry of the Arabian oryx, considering gender and age effects in two different geographic locations in Jordan.

## Materials and Methods

### Ethical approval

This study was approved by the Institutional Animal Use and Care Committee of Jordan University of Science and Technology (Approval no. 2014/0016).

### Study period and location

The study was conducted from May to August 2014.

Samples were collected from Arabian oryx kept at Al-Shaumari wildlife reserve, located in the eastern desert of Jordan, and Wadi Rum wildlife Reserve located in the southern desert of Jordan.

### Animals

A total of 49 Arabian oryx of various ages and sexes were used in this study. Animals were kept in animal reserves located in two geographical locations in Jordan. The first reserve is Al-Shaumari Wildlife Reserve, located in the eastern desert of Jordan, and the other is Wadi Rum Reserve, located in the southern desert of Jordan. Both locations are considered arid, with rainfall in the winter and cold weather, while in the summer, the weather is dry and hot. Natural shelters consisting of trees and bushes are available, as are isolated boxes and enclosures for animals to hide and rest. Water is supplied in the form of bonds. Feed is provided only during the dry season and consists of good-quality lucerne. During wet seasons, animals graze.

Veterinary care is provided to all animals through a regular veterinary consultant who visits the reserves on a regular basis. All animals appeared healthy and were subjected to complete physical examination before samples were collected. Animals were divided according to age (11 young – 1 year or less of age; and 33 adults – more than 1 year of age) and sex (16 males and 38 females).

### Sample collection

For examination and sample collection, animals were captured using a dart gun (Dan-Inject, Austin, TX 78754, USA) using medetomidine (Cayman Chemical, Michigan 48108, USA; 0.08 mg/kg i.m.), ketamine (Ketafast, Mumbai 400104, India; 2.2 mg/kg i.m.), and xylazine (Xylavet, Intervet, Isando 1600, South Africa; 0.01 mg/kg i.m. Animals were closely monitored during anesthesia and while being physically examined and monitored during whole blood withdrawal. Whole blood was withdrawn through jugular vein puncture using a vacutainer tube and needle and placed in plain and ethylenediaminetetraacetic acid (EDTA)-containing blood tubes. After completion of the examination and blood withdrawal, recovery from anesthesia was induced by reversal of xylazine using atipamezole (Antisedan, Zoites, Florham Park, New Jersey 07932, USA; 0.04 mg/kg i.m.). Animals were placed in a special padded box for recovery in a cool place and closely monitored until complete recovery, at this point, they were returned to their enclosures.

### Laboratory analysis

Whole blood in EDTA-containing tubes was used to determine total red blood cells (RBC), packed cell volume (PCV), hemoglobin (Hb) concentration, mean corpuscular volume, mean corpuscular hemoglobin (MCH), MCH concentration, and total leukocyte count (white blood cell [WBC]) using the ABC Vet Hematology Analyzer (ABX Diagnostics, Montpelier, France). For serum biochemical analyses, whole blood in plain tubes was placed in a centrifuge at 1500× *g* for 10 min to obtain serum. Serum samples were then used to determine glucose using the the glucose oxidase-phenol amino phenazone (GOP-PAP) method, total protein (biuret method), albumin (BCG method), and blood urea nitrogen (colorimetric method). All kits and reagents were obtained from Biolabo Reagents, Maizy, France. Calcium, magnesium, and phosphorus were determined using an Easylyte analyzer (Bedford, MA 01730, USA).

### Statistical analysis

The mean and standard error (SE) were generated among animal age and sex groups. Data were analyzed using SPSS software version 23 (IBM Statistics, USA). Before analyses, data were checked for normality using the Kolmogorov–Smirnov test. Outliers were identified and rejected using Tukey tests. A one-way analysis of variance was used to compare the effects of gender and age on the selected hematological parameters. Values were considered statistically significant at p < 0.05.

## Results

Table-1 shows the means and SE for selected hematology and serum biochemical parameters of normal adult and young Arabian oryx. There was a significant difference in RBC count, hematocrit, and serum glucose concentration between adult and young Arabian oryx. Red blood count and hematocrit were significantly lower in young oryx than in adults. Serum glucose concentrations were also significantly lower in young oryx.

**Table-1:** Means ± SE and 95% confidence limits for various hematology and serum biochemical parameters in normal adults and young Arabian oryx (*Oryx leukoryx*).

Parameters	Adults		Young	
	
	Mean ± SE	95% confidence limit	Mean ± SE	95% confidence limit
WBC (×10^3^ cells/µL)	13 ± 0.6	12–14	12 ± 0.8	10–14
RBC (×10^6^ cells/µL)	15 ± 3*	9–22	10 ± 0.6	9–11
Hemoglobin (g/dL)	16 ± 0.6	15–17	14 ± 0.5	13–16
PCV (%)	51 ± 2*	47–56	44 ± 2	39–49
MCV (fl)	46 ± 1	44–50	44 ± 0.7	42–45
MCH (pg)	18 ± 3	11–26	13 ± 1	10–16
MCHC (g/dL)	32 ± 0.4	31–33	33 ± 2	30–37
Platelets (×10^3^ cells/µL)	251 ± 30	189–314	256 ± 61	120–391
Total protein (g/L)	67 ± 1	64–71	61 ± 2	56–67
Albumin (g/L)	48 ± 1	45–51	44 ± 2	39–49
Glucose (mg/dL)	135 ± 8*	119–152	113 ± 6	99–127
Blood urea nitrogen (mg/dL)	23 ± 1	21–26	21 ± 3	15–27
Calcium (mg/dL)	8 ± 0.1	7–8	8 ± 0.3	7–9
Magnesium (mg/dL)	2 ± 0.1	2–3	2 ± 0.1	2–3
Phosphorus (mg/dL)	9 ± 0.3	8–10	9 ± 0.8	7–10

* p ≤ 0.05. RBC=red blood cell; Hb=Hemoglobin; PCV=Packed cell volume; MCV=Mean corpuscular volume; MCHC=Corpuscular hemoglobin concentration; MCH=Mean corpuscular hemoglobin; WBC=White blood cell, SE=Standard error

Table-2 shows the means and SE for selected hematology and serum biochemical parameters of normal male and female Arabian oryx. The table shows that RBC count is significantly higher in males than in females, while serum glucose concentration is significantly higher in females.

**Table-2:** Means ± SE and 95% confidence limits for various hematology and serum biochemical parameters in normal males and females Arabian oryx (*Oryx leukoryx*).

Parameters	Males		Females	
	
	Means ± SE	95% confidence limit	Means ± SE	95% confidence limit
WBC (×10^3^ cells/µL)	14 ± 0.5	12–16	12 ± 1	11–14
RBC (×10^6^ cells/µL)	17 ± 2*	4–31	13 ± 2	9–17
Hemoglobin (g/dL)	16 ± 0.4	13–18	16 ± 1	15–17
PCV (%)	52 ± 3	44–61	48 ± 3	45–52
MCV (fl)	44 ± 0.5	43–46	47 ± 2	44–50
MCH (pg)	14 ± 1	14–15	18 ± 1	10–27
MCHC (g/dL)	32 ± 1	31–32	33 ± 1	31–34
Platelets (×10^3^ cells/µL)	247 ± 14	160–333	255 ± 11	183–327
Total protein (g/L)	63 ± 4	58–68	67 ± 3	64–71
Albumin (g/L)	44 ± 2	41–48	49 ± 1	45–52
Glucose (mg/dL)	120 ± 4*	94–147	135 ± 4	119–150
Blood urea nitrogen (mg/dL)	25 ± 2	21–29	22 ± 3	20–25
Calcium (mg/dL)	8 ± 0.1	7–8	8 ± 0.7	8–9
Magnesium (mg/dL)	2 ± 0.1	2–3	2 ± 0.2	2–3
Phosphorus (mg/dL)	9 ± 0.5	8–10	9 ± 0.3	8–10

* p ≤ 0.05. RBC=Red blood cell; Hb=Hemoglobin; PCV=Packed cell volume; MCV=Mean corpuscular volume; MCHC=Corpuscular hemoglobin concentration; MCH=Mean corpuscular hemoglobin; WBC=White blood cell, SE=Standard error

## Discussion

Hematology and serum biochemical parameters are widely used to evaluate animals’ health and nutritional status. The analyses may shed light on certain pathological changes that might be occurring in different body systems due to disease or stress. The emphasis on establishing a reference range of normal baseline values cannot be overstated, especially regarding the evaluation of some wildlife species. The shy nature of such animal species and their resistance to capture make their clinical evaluation even more challenging. The Arabian oryx is poorly adapted to live in captivity or in small enclosures and often expresses a severe, stressful response to handling. A combination of xylazine and ketamine has been used to immobilize oryx for many years to allow clinical evaluation and sample collection [13–16]. In this study, we used a mixture of medetomidine, xylazine, and ketamine with great success. The mixture works immediately (the animal is down within 2–3 min) and lasts almost 30 min with smooth induction and recovery. Many researchers have suggested that the immobilization method may affect hematology and serum biochemical parameters [13–16]. Therefore, it is essential when reporting normal values for a wildlife species to discuss the method of immobilization used for future comparison. Stress from capture in wild animals can affect both WBCs and RBCs, PCV, and Hb due to catecholamine release and splenic contraction, respectively [17]. In agreement with previously reported data, WBC count, RBC count, PCV, and Hb were higher than those of other domestic animal species [18–20]. In the serum biochemical analysis, glucose was the only elevated value, most likely due to stress. This effect was more obvious in adult females.

When compared between adults and young oryx, there was a significant difference in RBC count, hematocrit, and serum glucose concentration. Red blood cell count, hematocrit, and serum glucose concentrations were significantly lower in young oryx than in adults. This may indicate that young animals may tolerate capture better than their adult parents. This may also indicate that the spleen reserves of RBCs are not as large as they are in adult animals [18–20].

In this study, we report a safe, fast, and effective combination to immobilize Arabian oryx (*O. leukoryx*) using medetomidine-xylazine-ketamine using a dart gun. The normal reference range of hematology and serum biochemical analysis in adult and young males and females immobilized using this combination is reported for the first time. These data can be used for health control and diagnosis of diseases in this species after immobilization using this chemical mixture.

## Conclusion

Various hematology and serum biochemistry parameters were determined in apparently normal Arabian oryx. Knowledge of these data could prove vital in the clinical evaluation of the health status of this wildlife species.

## Authors’ Contributions

AE: Conceived and designed the study, collected the samples, and performed laboratory analyses. ZBI: Analyzed and interpreted the data and edited the manuscript. Both authors have read, reviewed, and approved the final manuscript.
